# Assessing Evidence‐Based Practice Competence in Nurse Leadership Roles in Spain

**DOI:** 10.1155/jonm/9254720

**Published:** 2026-05-18

**Authors:** Gabriel Segura-López, Pedro Simón Cayuela-Fuentes, César Leal-Costa, Serafín Fernández-Salazar, José Antonio Vera-Pérez, Antonio Jesús Ramos-Morcillo, María Ruzafa-Martínez

**Affiliations:** ^1^ Faculty of Nursing, Department of Nursing, University of Murcia, Murcia, Spain, um.es; ^2^ Andalusian Health Service, AGS Northeast Jaen, Úbeda, Spain, juntadeandalucia.es

**Keywords:** competency, EBP-COQ Prof, evidence-based practice, management, nurse, nurse managers

## Abstract

**Objective:**

This study evaluated the competency of nursing leaders and managers in Spain regarding evidence‐based practice (EBP) and identified factors that affect its implementation in clinical environments.

**Background:**

EBP is essential for enhancing healthcare quality; however, there are still gaps between theoretical knowledge and its practical application. Nursing leaders are crucial in promoting EBP, yet their competence levels and obstacles have not been thoroughly examined in Spain.

**Material and methods:**

A cross‐sectional study surveyed 159 nurse managers across 16 autonomous communities in Spain, utilizing the validated EBP‐COQ Prof tool. The questionnaire assessed four competency domains—attitude, knowledge, skills, and utilization—using a 1 to 5 Likert scale. Predictors of EBP competency, including training, mentorship, and organizational affiliation, were analyzed using multivariate linear regression.

**Results:**

Participants demonstrated a strong overall EBP competence, with an average total score of 143.77 out of 175. The highest scores were in the attitude domain (36.26/40) and knowledge (43.09/55), whereas utilization scores were relatively lower (37.57/50). This indicates that applying EBP in practice still lags behind positive attitudes and knowledge. Key factors predicting higher EBP competence included EBP training, consistent reading of scientific literature, mentoring nursing students, and working in a BPSO center. These results also imply that organizational constraints may continue to impede the integration of EBP competence into routine managerial practice.

**Conclusions:**

Spanish nurse managers possess strong EBP knowledge but continue to encounter difficulties in applying it clinically. The study highlights four adjustable factors of EBP competency that could guide focused interventions.

**Implications for Nursing Management:**

Healthcare institutions should implement (1) training programs for addressing knowledge‐to‐practice gaps, especially for nonspecialist managers; (2) mentorship systems pairing EBP‐competent leaders with novices; (3) protected time for EBP activities; and (4) expanded BPSO accreditation to institutionalize evidence‐based care. Future initiatives should include EBP metrics in performance evaluations, and research should explore factors such as leadership styles and the impact of resource allocation on patient outcomes through longitudinal designs.

## 1. Introduction

The healthcare profession increasingly focuses on evidence‐based practice (EBP) as a cornerstone for quality decision‐making and improving patient outcomes [1]. EBP integrates the highest level of research evidence with clinical expertise, patient values, and preferences in making healthcare decisions [2]. Scientifically driven evidence, not tradition or intuition, grounds these interventions and policies.

Recent international research continues to underscore the complex, multidimensional nature of EBP competence among various nursing groups. Studies in Palestine reveal that both practicing nurses and nursing students often hold positive attitudes toward EBP, yet they face limitations in knowledge, skills, and practical application, as well as barriers to implementation [3, 4]. These results highlight the importance of assessing EBP competence not only as an educational goal but also as a professional and organizational challenge across different healthcare environments.

In nursing, leaders and managers play a crucial role in cultivating a culture of EBP within their organizations [5]. They are responsible for establishing an environment that encourages the adoption of evidence‐based protocols and guidelines, while also fostering critical thinking and a commitment to lifelong learning among their teams [6]. The success of healthcare delivery largely hinges on effectively translating research evidence into clinical practice, where nurse leaders and managers act as vital links between research and practice [7].

Assessing the EBP competency levels of nursing executives and managers in Spain is vital for recognizing strengths and opportunities for improvement in the healthcare system. This insight can guide the development of targeted interventions to enhance their skills and expertise in EBP. Developing EBP competencies among nursing leaders can lead to higher quality care, greater patient safety, and more efficient resource utilization. A thorough grasp of EBP enables nurse executives to thoughtfully analyze new research and effectively integrate proven findings into clinical practice, thereby enhancing patient care outcomes [8].

Nonetheless, fragmented practices and ongoing gaps between evidence and application frequently lead to early failures in EBP initiatives, posing significant implementation challenges. For successful implementation, adopting a multidimensional approach that considers individual and organizational factors is essential. Nurses in leadership positions often face difficulties implementing EBPs due to colleague resistance, resource limitations, and insufficient management support [9]. Tackling these issues requires a comprehensive strategy that includes leadership training, the development of clear and effective communication methods, and the promotion of a workplace that values and encourages EBP [10]. Furthermore, nurse leaders must excel in fostering collaboration within multidisciplinary teams, navigating complex organizational structures, and advocating for policies that bolster EBP [11].

Strong leadership skills are essential for nurse leaders and managers to thrive, regardless of their positions in clinical practice, administration, or executive leadership [12]. Those with enhanced leadership abilities are more adept at navigating the complexities of healthcare administration, motivating their teams, and promoting EBP in their environments [13]. Given the rising demands on healthcare systems, it is crucial to equip nurse leaders with the necessary skills to foresee and tackle future challenges. The effective use of these competencies significantly impacts their capacity to enhance care quality, boost patient satisfaction, and improve overall healthcare outcomes [12, 14]. The rapidly evolving landscape of health knowledge and technological advancements necessitates that nursing leaders engage in ongoing professional development to stay proficient and guide their teams in embracing innovative EBP strategies [15].

Various factors influence the extent of EBP competency in nursing leaders and managers. These factors include their educational background, the range and depth of their clinical experience, and access to continuous professional development opportunities [16]. Cognitive agility, defined by strong critical thinking, problem‐solving skills, and analytical reasoning, allows leaders to assess complex research evidence and synthesize it effectively [17].

Some researchers emphasize that nursing managers’ ability to connect evidence use directly with improvements in care quality and patient safety is essential in fostering a culture of EBP. Moreover, these managers need diverse educational, instructional, and research‐related skills to support clinical teams in embracing and implementing EBPs. Nonetheless, these initiatives rely more on informal relationships, role modeling, and experiential methods to validate evidence, rather than on structured systems or formalized frameworks [18, 19].

Nurse managers play a crucial role in bridging research and clinical practice. They ensure that scientific findings are effectively shared and support the organization needed for integrating this knowledge into everyday patient care. Positioned strategically within multidisciplinary teams, they can lead systemic changes in healthcare, fostering the adoption of innovative strategies based on the best available evidence. Their broad perspective allows them to grasp the effects of evidence‐based interventions and policies, ensuring decisions are advantageous for healthcare professionals and patients [20, 21].

Studies conducted in Spain resonate with international literature, revealing shared obstacles to advancing EBP, including limited time and insufficient training in the necessary knowledge and skills [22, 23]. Although prior research in Spain has investigated EBP among nurses [24, 25], it lacks specific national data on nurse managers and leaders. Additionally, it has not thoroughly examined the individual and organizational factors associated with EBP competence within this group. This gap is significant in the Spanish context because of the vital role nurse managers play in fostering evidence‐informed clinical and organizational decisions.

This study aimed to (1) evaluate the level of EBP competence among nurse managers and leaders in Spain with the EBP‐COQ Prof© questionnaire and (2) explore the sociodemographic, professional, and scientific information access factors linked to this competence in nursing management.

## 2. Method

### 2.1. Design

A Spanish cross‐sectional study, called the #Evidencer study, was conducted in 2020 [24]. Although collected in 2020, the data remain highly relevant because the fundamental factors influencing EBP have remained stable in healthcare systems, underscoring the current significance of these findings.

### 2.2. Participants

Using nonprobabilistic, stratified sampling by autonomous community, participants were recruited from nurses working in public health centers affiliated with the Spanish National Health System (NHS). The inclusion criteria for the overall campaign were as follows: being an actively employed nurse in an NHS‐affiliated public healthcare center, having at least 1 year of professional experience, and working in hospital or primary care settings under any type of contract. From the total campaign sample of 2942 nurses, an analytic subsample of 159 managerial nurses was selected for this study.

For the purposes of this study, “managerial nurses” were defined as registered nurses who reported holding a formal leadership or management position within their healthcare organization, such as ward/unit managers, supervisors, coordinators, middle managers, or nursing directors, and whose responsibilities included staff supervision, organization of care, resource management, and/or clinical decision‐making. Subsampling was based on professional role identification within the broader survey dataset and did not involve additional exclusion criteria beyond those established for the original campaign. Although stratification by autonomous community guided recruitment in the national survey to improve territorial representation, no weighting procedure was applied specifically to this managerial subsample.

### 2.3. Variables and Instruments

A digital form was created that encompassed the following variables:

Sociodemographic: age, sex, and civil status.

Professional: year of completion of studies, years of professional experience, work environment (rural or urban), work situation, educational level achieved (other bachelor’s degree, master’s degree and Ph.D. degree), training in EBP, number of articles read in the last month, tutoring of nursing students, working in a Best Practice Spotlight Organization (BPSO®) center, health centers participating in the international program for the implementation of clinical practice guidelines developed by the Registered Nurses’ Association of Ontario [26].

Related to access to scientific information: use of the Internet and social networks, frequency of use, use of Twitter, health blogs, most frequent places of access to the Internet, and access to the Internet in the workplace.

EBP competence was assessed using the Evidence‐Based Practice Competence in Professionals (EBP‐COQ Prof©) questionnaire in a validated Spanish context, ensuring validity and reliability [27]. It was designed based on the EBP competency framework for general care nurses outlined by Melnyk et al. [28], defining competency as “the nurse’s ability to integrate cognitive, affective, and psychomotor skills in the provision of nursing care [29].” This definition encompasses both potential behavior (including attitudes, knowledge, and skills) and actual behavior (the application of EBP in clinical settings) [30]. The questionnaire contains 35 items rated on a 1 to 5 Likert scale, categorized into four dimensions: attitude (8 items, scoring from 8 to 40), knowledge (11 items, ranging from 11 to 55), skills (6 items, spanning 6–30), and utilization (10 items, covering 10–50). The EBP proficiency score ranges from 35 to 175 points, indicating that a higher score reflects greater proficiency. Although the EBP‐COQ Prof© has been previously validated in the Spanish context, internal consistency was also examined in the present sample to provide reliability evidence for this study population. The internal consistency of the EBP‐COQ Prof© was satisfactory, with a Cronbach’s alpha of 0.923 for the total scale. The coefficients for the subdimensions were 0.781 for attitude, 0.938 for knowledge, 0.738 for skills, and 0.805 for utilization.

### 2.4. Data Collection Procedure

The data collection was conducted online between January and March of 2020, utilizing a national collaborative campaign called #Evidencer.

The campaign solicited the engagement of nurses nationwide via social media platforms, professional schools, trade unions, and scientific organizations.

A four‐page online questionnaire was meticulously designed, incorporating the variables delineated in the preceding section, and made accessible through the Google Forms platform. The estimated completion time was approximately 15 minutes.

### 2.5. Data Analysis

A univariate descriptive analysis was conducted, incorporating measures of central tendency for the quantitative sociodemographic variables and the EBP‐COQ Prof© dimensions. Additionally, absolute frequencies and percentages were calculated for the categorical variables. A bivariate analysis was conducted on the dimension scores and total score of the EBP‐COQ Prof©, along with sociodemographic and professional variables, using *t*‐tests for independent variables, one‐way ANOVA, and Pearson’s correlation. Only participants with complete responses to all questionnaire items were included in the analyses; therefore, no missing data were present and no imputation procedures were required. For the multivariate analysis, multiple linear regression models were fitted to examine the association between the independent variables and the EBP‐COQ Prof© total score and each of its dimensions (attitude, knowledge, skills, and utilization). These outcomes were treated as continuous variables because they were derived from summated scores of multiple Likert‐type items within a validated instrument, an approach commonly accepted in psychometric and health research. Before fitting the regression models, the assumptions of linear regression were assessed. Normality was examined through inspection of residual distributions and normal probability plots; homoscedasticity and linearity were evaluated using residual‐versus‐fitted plots; independence of errors was assessed with the Durbin–Watson statistic; and multicollinearity was examined using tolerance values and variance inflation factors (VIF). No substantial violations of these assumptions were detected. The forward stepwise sequential method was applied, guided by the criteria of F probability for entry ≤ 0.05 and exit > 0.10. This approach was chosen because the analysis was exploratory and aimed to identify the most relevant predictors of EBP competence from a broad set of candidate variables. In the absence of a sufficiently established theoretical model to support hierarchical entry and given the sample size relative to the number of potential predictors, a forward stepwise procedure was considered appropriate to obtain parsimonious and interpretable models. A significance level of 5% (*p* ≤ 0.05) was established for the statistical analysis. The analyses were conducted using the SPSS 26.0 program.

### 2.6. Ethical Considerations

The study received approval from the Ethics Committee of the University of Murcia (ID: 2540/2019). Professionals were invited to participate voluntarily via an online questionnaire. Participants were informed of the study’s objectives, emphasizing that their involvement was anonymous and that completing the questionnaire constituted their consent to participate.

## 3. Results

The final sample consisted of 159 managerial nurses from 16 autonomous communities in Spain. Table 1 presents the key characteristics of the participants, who had an average age of 46.35 years (SD = 8.61). Among them, 71.1% (*n* = 113) were female, with an average work experience of 23.58 years (SD = 9.08). Furthermore, 76.7% of the participants were employed in urban areas, and 133 individuals (83.6%) held permanent contracts. Regarding education, 119 nurses (74.8%) completed a master’s degree. Most of these nurses had also undergone EBP training (95.6%) and frequently used the Internet to gather scientific information (85.5%).

**TABLE 1:** Descriptive statistics of the sample characteristics.

Variables	*M*	SD
Age (years)	46.35	8.61
Years since the completion of the nursing degree (years)	25.17	9.14
Professional experience (years)	23.58	9.08

	***n***	**%**

Sex		
Male	46	28.9
Female	113	71.1
Civil status		
Single	21	13.2
Married	122	76.7
Divorced	14	8.8
Widow	2	1.3
Other bachelor’s degree		
Yes	34	21.4
No	125	78.6
Master degree		
Yes	119	74.8
No	40	25.2
Ph.D. degree		
Yes	27	17.0
No	132	83.0
Nursing specialty		
Yes	12	7.5
No	147	92.5
Employment status		
Eventual	6	3.8
Interim	20	12.6
Fixed	133	83.6
Labor dedication		
Full time	155	97.5
Part time	4	2.5
Work environment		
Urban (> 50,000 inhabitants)	122	76.7
Urban (between 10,000 and 50,000 inhabitants)	30	18.9
Rural (< 10,000 inhabitants)	7	4.4
Workspace		
Hospital care	134	84.3
Primary care	14	8.8
Social and Health Center	3	1.9
Private Domain	8	5
EBP training		
None	7	4.4
Less than 40 h	26	16.4
Between 40 and 150 h	56	35.2
More than 150 h	70	44.0
Number of articles read in the last month		
None	9	5.7
Between 1 and 3	51	32.1
Over 3	99	62.3
Working at a BPSO center		
Yes	70	44
No	89	56
Undergraduate nursing student tutoring		
Yes	74	46.5
No	85	53.5
Use of the Internet and other digital tools to access scientific information		
Yes	136	85.5
No	23	14.5
Using Twitter to access scientific information		
Yes	114	71.7
No	45	28.3
Use of health blogs to access scientific information		
Yes	129	81.1
No	30	18.9
Access to the Internet at work		
Yes	154	96.9
No	5	3.1
The place where they most frequently access the Internet to consult information		
Home	104	65.4
Work	55	34.6
Frequency of use of the Internet and other digital tools to access scientific information		
Never	11	6.9
Occasionally	28	17.6
Monthly	5	3.1
Weekly	43	27.0
Daily	72	45.3
Autonomous Community		
Andalucía	36	22.6
Aragón	3	1.9
Asturias	7	4.4
Cantabria	4	2.5
Castilla‐La Mancha	2	1.3
Castilla y León	10	6.3
Cataluña	11	6.9
Comunidad Valenciana	12	7.5
Extremadura	3	1.9
Galicia	6	3.8
Islas Baleares	5	3.1
Islas Canarias	7	4.4
Madrid	21	13.2
Murcia	12	7.5
Navarra	8	5.0
País Vasco	12	7.5

*Note:* M: mean.

Abbreviations: BPSO, Best Practice Spotlight Organization; SD, standard deviation.

The average score for the EBP competency level was 143.77 (SD = 14.45). For each dimension of the EBP‐COQ Prof©, the results indicated a mean score of 36.26 (SD = 2.24) for EBP attitude, 43.09 (SD = 7.72) for EBP knowledge, 24.85 (SD = 3.06) for EBP skills, and 37.57 (SD = 5.36) for EBP utilization. These results suggest that although nurse managers reported highly favorable attitudes toward EBP, utilization scores were comparatively lower, suggesting a possible gap between these attitudes and their application in practice.

Table 2 displays the categorical variables that reveal a statistically significant relationship with the dimensions of EBP competence based on the bivariate results. Relationships were observed between some dimensions from the EBP‐COQ Prof© questionnaire and levels of education (master’s and Ph.D. degrees), EBP training, reading scientific articles, frequency of Internet use, and other digital tools for accessing scientific information, being a nursing student mentor, and working in a BPSO® center.

**TABLE 2:** Bivariate analysis of sociodemographic and professional variables with the dimensions and total of EBP‐COQ‐Prof.

Variables	*n*	EBP attitude			EBP knowledge			EBP skills		EBP utilization				EBP total		
		*r*	*p* value		*r*	*p* value		*r*	*p* value	*r*		*p* value		*r*	*p* value	
Age (years)	159	−0.014	0.862		0.020	0.799		−0.10	0.902	0.035		0.663		0.020	0.807	
Years since the completion of the nursing degree (years)	159	−0.027	0.734		0.026	0.743		0.000	0.998	0.062		0.439		0.033	0.682	
Professional experience (years)	159	−0.025	0.750		0.045	0.575		0.030	0.708	0.092		0.250		0.060	0.450	

	***n***	***M***	**SD**	***p* value**	***M***	**SD**	***p* value**	***M***	**SD**	***p* value**	***M***	**SD**	***p* value**	***M***	**SD**	***p* value**

Male	46	38.00	2.59	0.357	43.46	8.10	0.702	25.52	3.22	0.08	38.65	5.22	0.105	145.63	15.43	0.303
Female	113	38.36	2.09		42.94	7.60		24.58	2.97		37.13	5.38		143.02	14.04	
Other bachelor’s degree																
Yes	34	38.47	2.00	0.534	43.12	7.75	0.980	24.85	3.65	0.996	38.21	5.59	0.454	144.65	16.48	0.692
No	125	38.20	2.31		43.08	7.75		24.86	2.90		37.40	5.31		143.54	13.91	
Master’s degree																
Yes	119	38.26	2.19	0.980	44.71	6.33	< 0.001	25.25	2.72	0.005	37.97	5.00	0.103	146.20	12.19	< 0.001
No	40	38.25	2.43		38.25	9.39		23.68	3.72		36.38	6.24		136.55	18.03	
Nursing specialty																
Yes	12	37.42	2.27	0.177	42.67	7.70	0.845	25.25	1.22	0.338	36.25	5.51	0.376	141.58	8.88	0.587
No	147	38.33	2.23		43.12	7.75		24.82	3.17		37.68	5.35		143.95	14.82	
Ph.D. degree																
Yes	27	38.56	2.34	0.451	47.85	5.93	< 0.001	25.81	3.31	0.074	37.78	6.16	0.828	150.00	14.93	0.014
No	132	38.20	2.23		42.11	7.70		24.66	2.99		37.53	5.21		142.50	14.07	
Training on EBP																
(1) None	7	37.57	3.05	0.804	33.00^4^	8.47	< 0.001	20.57^4^	3.78	< 0.001	32.86	6.04	0.029	124.00^4^	15.61	< 0.001
(2) < 40 h	26	38.31	1.74		38.42^4^	7.75		24.62	3.15		36.15	5.70		137.50^4^	13.81	
(3) 40–150 h	56	38.16	2.23		42.09^4^	6.63		24.48	2.78		37.82	5.13		142.55^4^	12.52	
(4) > 150 h	70	38.39	2.36		46.63^1,2,3^	6.45		25.67^1^	2.80		38.37	5.12		149.06^1,2,3^	13.39	
Number of articles read in the last month																
(1) 0	9	36.22	4.09	0.019	33.22^2,3^	6.48	< 0.001	22.89^3^	2.37	< 0.001	31.89^3^	4.17	< 0.001	124.22^2,3^	8.64	< 0.001
(2) 1 to 3	51	38.35	1.78		40.04^1,3^	7.56		23.65^3^	3.12		36.02^3^	5.18		138.06^1,3^	13.71	
(3) > 3	99	38.39	2.18		45.56^1,2^	6.61		25.66^1,2^	2.82		38.89^1,2^	5.03		148.49^1,2^	12.66	
Use of digital tools to access scientific information																
Yes	136	38.30	2.24	0.566	43.30	7.53	0.399	24.87	2.94	0.903	37.46	5.46	0.507	143.93	14.21	0.747
No	23	38.00	2.32		41.83	8.87		24.78	3.80		38.26	4.81		142.87	16.14	
Access to the Internet at work																
Yes	154	38.27	2.26	0.644	43.18	7.73	0.431	24.86	3.07	0.851	37.66	5.40	0.277	143.97	14.52	0.349
No	5	37.80	1.92		40.40	7.83		24.60	3.05		35.00	3.46		137.80	12.03	
Frequency of Internet use and other digital tools for accessing scientific information																
(1) Never	11	37.00	3.52	0.387	36.73	8.76	0.007	23.73	4.03	0.467	36.82	5.60	0.483	134.27	17.09	0.041
(2) Occasionally	28	38.21	2.35		40.61	9.29		24.39	3.59		36.04	6.00		139.25	17.75	
(3) Monthly	5	38.60	1.14		43.60	2.51		23.80	0.84		37.80	1.92		143.80	3.77	
(4) Weekly	43	38.23	2.17		43.49	7.41		25.16	3.02		38.30	5.82		145.19	13.67	
(5) Daily	72	38.46	2.05		44.75	6.68		25.10	2.79		37.83	4.92		146.14	12.79	
Nursing student mentor																
Yes	74	38.47	1.93	0.260	44.30	6.99	0.065	25.54	2.97	0.008	38.89	5.26	0.003	147.20	13.66	0.005
No	85	38.07	2.48		42.04	8.20		24.26	3.04		36.42	5.21		140.79	14.54	
Working in a BPSO center																
Yes	70	38.74	1.56	0.010	43.57	7.43	0.486	25.26	2.99	0.143	39.40	4.78	< 0.001	146.97	12.67	0.011
No	89	37.88	2.61		42.71	7.96		24.54	3.10		36.13	5.38		141.26	15.32	

*Note:* M = Mean; ^1,2,3,4 and 5^the category of nurses with which it has statistically significant differences (*p* < 0.05) in the pairwise analysis of the Games–Howell post hoc comparison test.

Abbreviation: SD, standard deviation.

No significant correlations were observed between the quantitative sociodemographic variables and the dimensions from the EBP‐COQ Prof© questionnaire (Table 2).

**FIGURE 1:**
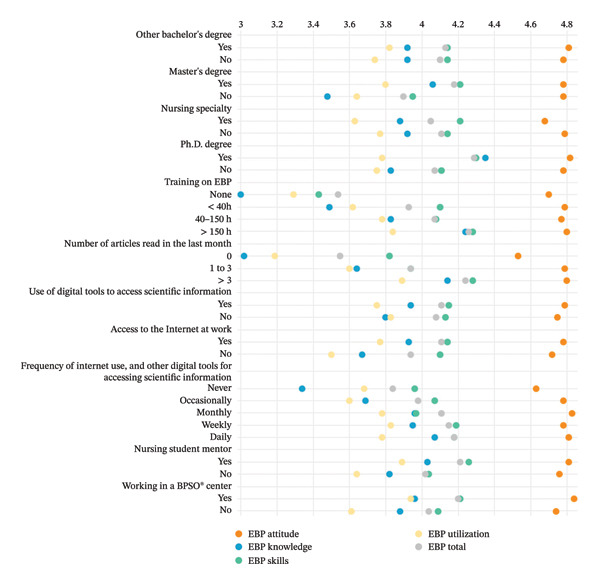
Nurses’ EBP competence levels by key variables (weighted Likert scores 1–5).

Figure 1 illustrates the weighted scores for sociodemographic, professional, and access to scientific information variables regarding the various dimensions and total EBP‐COQ Prof©, which is based on a 1 to 5 Likert‐scale response format. This visualization enhances the clarity of nurses’ responses, showcasing differences in EBP competence (total score) and its subdimensions—attitude, knowledge, skills, and utilization—driven by various influencing factors. By standardizing responses on a comparable scale, this figure offers an intuitive grasp of how these variables connect with nurses’ self‐reported EBP competence. The weighted scores reflect response distribution, delivering a more precise portrayal of trends and differences in EBP proficiency across diverse nurse subgroups.

Multivariate analysis highlights the final variables included in the fitted models and the effect magnitudes linked to the four dimensions and the EBP‐COQ Prof© total score (Table 3). The most influential factors on the total score and all dimensions of the EBP‐COQ Prof© are the number of articles read in the prior month and EBP training. Mentoring nursing students also contributes positively to skills and utilization, along with the overall EBP‐COQ Prof© score. Higher educational levels (master’s and doctorate) correlate with the knowledge dimension. At the same time, employment at a BPSO® center is related to EBP attitudes, EBP utilization dimensions, and the total EBP‐COQ Prof© score.

**TABLE 3:** Multiple linear regression models categorizing dimensions and overall competence according to the EBP‐COQ Prof.

Dependent variable	Independent variables	Unstandardized (*B*)	Standardized *β*	*p*	*R* ^2^	Durbin–Watson
Total EBP competence	Number of articles read in the last month	7.638	0.318	< 0.001	0.318	2.06
	Training on EBP	4.299	0.257	0.001		
	Nursing student mentor	5.914	0.205	0.003		
	Working in a BPSO Center	5.154	0.178	0.008		

EBP attitude	Working in a BPSO Center	0.866	0.192	0.015	0.031	1.93

EBP knowledge	Training on EBP	2.637	0.295	< 0.001	0.348	1.93
	Number of articles read in the last month	3.148	0.245	0.001		
	Master’s degree	3.162	0.178	0.011		
	Ph.D. degree	3.329	0.162	0.015		

EBP skills	Number of articles read in the last month	1.116	0.219	0.009	0.155	1.97
	Nursing student mentor	1.145	0.187	0.013		
	Training on EBP	0.712	0.201	0.016		

EBP utilization	Number of articles read in the last month	2.793	0.313	< 0.001	0.241	2.18
	Working in a BPSO Center	3.309	0.307	< 0.001		
	Nursing student mentor	2.336	0.218	0.002		

Abbreviation: BPSO, Best Practice Spotlight Organization.

## 4. Discussion

This study provides the first national assessment of EBP competence among Spanish nurse managers using the validated EBP‐COQ Prof© instrument [27]. Furthermore, it explores the relationships between sociodemographic and professional factors and the questionnaire’s various dimensions. Overall, the findings indicate that nurse managers in Spain demonstrate a high level of EBP competence, with total scores exceeding those previously reported for the general nursing population in Spain [22]. Among the four competency domains, attitudes toward EBP were particularly strong, whereas EBP utilization showed comparatively lower scores, highlighting a persistent gap between theoretical competence and practical implementation.

The study also highlighted several modifiable factors that influence EBP competence, such as EBP training, consistent reading of scientific literature, mentoring nursing students, and working in BPSO® centers. These findings emphasize the importance of both individual professional growth and organizational environment in promoting evidence‐based nursing leadership.

The #Evidencer project involved 159 Spanish nurse managers, averaging 46 years old, with 71% of them being female. They represented 16 autonomous communities, and this profile is similar to that of the nurses working in the Spanish NHS [31].

The elevated overall EBP competence observed among Spanish nurse managers can be partially explained by the sample’s professional profile, characterized by extensive clinical experience, high levels of postgraduate education, and widespread participation in EBP training. This aligns with previous international research, demonstrating that advanced education and continuous professional development are key determinants of EBP knowledge and skills among nursing professionals [6, 18, 22].

The notably high scores in the attitude domain indicate that nurse managers are highly committed to EBP, supporting evidence that leadership involvement is a key to creating and maintaining an EBP culture in healthcare organizations [32]. This aligns with systematic review, demonstrating that leaders who prioritize evidence‐based decision‐making positively impact care quality, staff engagement, and organizational results [2].

Although participants demonstrated very positive attitudes and solid knowledge of EBP, their utilization scores were relatively lower, indicating that the actual application of EBP in practice is still behind their attitudinal readiness and understanding. This gap highlights ongoing structural and organizational barriers—such as limited time, competing administrative duties, and lack of organizational support—that impede the routine application of EBPs [33, 34]. Such discrepancies between EBP competence and actual implementation have been observed across various healthcare systems, indicating that these issues are systemic rather than context‐specific [19, 35].

This interpretation is also supported by recent European evidence. In the Portuguese context, formal nursing leaders identified organizational conditions, leadership support, and resource‐related factors as key determinants of EBP implementation. Likewise, recent evidence indicates that nurses′ adoption of EBP is strongly influenced by professional practice environments, management and leadership, teamwork, communication, and organizational infrastructure [34, 36]. Taken together, these findings suggest that the attitude–utilization gap observed in the present study is not unique to the Spanish context, but rather reflects broader cross‐cultural challenges in embedding EBP within nursing leadership and daily clinical management.

Multivariate analysis revealed that reading scientific articles and EBP training were the strongest predictors of overall EBP competence, corroborating prior studies emphasizing ongoing engagement with research literature as essential for maintaining EBP proficiency [1, 8]. Furthermore, the positive association between mentoring nursing students and higher scores in skills and utilization highlights the value of teaching roles in reinforcing reflective practice, self‐efficacy, and applied competence, consistent with earlier evidence on experiential learning and professional role modeling [24, 37, 38].

From an implementation science perspective, these findings can be interpreted through frameworks such as i‐PARIHS, which emphasize the dynamic interaction between evidence, facilitation, and context as key determinants of successful EBP implementation [39]. In this regard, the association between working in a BPSO® center and higher levels of EBP utilization and more favorable attitudes underscores the pivotal role of organizational infrastructure in enabling evidence uptake. This finding is consistent with previous research indicating that structured implementation frameworks, strong leadership support, and formalized processes—such as those embedded within the BPSO® program—facilitate the sustained integration of EBP into clinical and managerial practice [18, 26].

Future research should employ longitudinal and intervention‐based approaches to assess how targeted strategies—such as structured mentorship, leadership development programs, and allocated time for EBP activities—impact managerial skills and patient outcomes. Additionally, qualitative research exploring nurse managers’ personal experiences could offer more profound understanding of the contextual and relational factors impacting EBP adoption. Investigating the connection between nurse managers’ EBP competence and outcomes such as staff retention, job satisfaction, and quality measures would also help highlight the strategic importance of evidence‐based nursing leadership in healthcare systems.

This study provides a national evaluation of EBP competence among Spanish nurse managers using the validated EBP‐COQ Prof© instrument, which assesses four key competency domains. The inclusion of participants from 16 autonomous communities broadens the study’s geographical scope and provides a comprehensive overview of nursing management in the Spanish public healthcare context. However, several limitations should be acknowledged. First, the cross‐sectional design precludes causal inferences. Second, the use of nonprobabilistic and voluntary sampling may have introduced selection bias, as managers with greater interest in EBP, stronger academic profiles, or higher prior exposure to EBP may have been more likely to participate. As a result, the observed competence levels may overestimate the actual EBP competence of nurse managers in Spain. Third, although recruitment was stratified by autonomous community in the parent study, the managerial subsample was not weighted and therefore should not be considered statistically representative of all Spanish nurse managers. Fourth, the exclusive use of self‐reported measures may have introduced common‐method bias and social desirability bias. Finally, the potential presence of ceiling effects, particularly in the attitude dimension, may have limited score variability and reduced the instrument’s ability to discriminate between participants in this domain. Relevant contextual variables such as leadership style, institutional culture, and resource availability were also not directly measured.

## 5. Conclusion

This study shows that Spanish nurse managers possess a strong foundational competence in EBP, particularly in the domains of attitude and knowledge. However, comparatively lower utilization scores indicate that important barriers remain in translating EBP competence into routine managerial and clinical practice. Training in EBP, engagement with scientific literature, mentoring roles, and affiliation with BPSO® centers emerged as key determinants of competency.

These findings suggest that efforts to strengthen EBP in nursing management should move beyond individual training alone and include organizational and policy‐level strategies. In particular, EBP should be more explicitly integrated into leadership development programs, postgraduate nursing management curricula, and continuing professional education for nurse managers. In addition, healthcare organizations and policymakers should consider incentives to expand BPSO®‐type initiatives and other structured implementation models that create supportive environments for evidence uptake. Such actions may help bridge the gap between positive attitudes toward EBP and its effective use in practice, ultimately contributing to higher quality care and more sustainable healthcare systems.

## Funding

This study was supported by a grant from the Ministerio de Ciencia e Innovación, reference number: PID2019‐106545GA‐I00/AEI.

## Ethics Statement

The study was approved by the Ethics Committee of the University of Murcia (ID: 2540/2019).

## Conflicts of Interest

The authors declare no conflicts of interest.

## Data Availability

The data that support the findings of this study are available upon request from the corresponding author. The data are not publicly available due to privacy or ethical restrictions.
